# Ohio

**Published:** 1897-03

**Authors:** J. C. Meyer

**Affiliations:** Member of the Board


*Ohio.* Nothing in this Act shall be construed to prohibit any veterinary advice or service in cases of emergency if rendered by a person not entitled to practice under this Act; nor shall it apply to animal castration and dehorning of cattle.—*Dr. J. C. Meyer, Jr., Member of the Board.*

